# A Case Report of Acute Transient Encephalopathy Following a Trans-esophageal Echocardiography

**DOI:** 10.7759/cureus.18580

**Published:** 2021-10-07

**Authors:** Wasey Ali Yadullahi Mir, Dhan B Shrestha, Vijay Ketan Reddy, Suman Gaire, Larissa Verda

**Affiliations:** 1 Department of Internal Medicine, Mount Sinai Hospital, Chicago, USA; 2 Department of Medicine, Mount Sinai Hospital, Chicago, USA; 3 Department of Emergency Medicine, Palpa Hospital, Palpa, NPL

**Keywords:** benzocaine, local anesthetics, methemoglobinemia, transesophageal echocardiography, lidocaine, cyanosis, hypoxia

## Abstract

Methemoglobinemia is caused due to an increase in methemoglobin in the blood, impairing oxygen transfer to tissues. Acquired methemoglobinemia is caused by various drugs like local anesthetics, antibiotics, nitrates, nitrites, and food additives. We present a case of a 73-year-old male who presented with cyanosis, altered mental status, and hypoxia following transesophageal echocardiography. Arterial blood gas analysis revealed methemoglobinemia. He had been given topical lidocaine and benzocaine spray before the procedure. He improved after the administration of methylene blue. The case highlights the importance of considering methemoglobinemia in patients presenting with cyanosis, altered mental status, and hypoxia after endoscopic procedures.

## Introduction

Methemoglobinemia is a rare condition of the blood associated with an increase in the proportion of methemoglobin, which affects the oxygen-carrying capacity of the blood. There are two types of methemoglobinemia, congenital and acquired. Congenital/inherited methemoglobinemia is caused by a genetic deficiency of an enzyme cytochrome B5 reductase or a hemoglobin protein defect (Hemoglobin M disease). The acquired form is associated with exposure to certain chemicals/medications like local anesthetics (benzocaine, prilocaine, lidocaine); antibiotics (dapsone, sulfonamides, chloroquine); nitrates (salts, nitroglycerin); nitrites (nitroprusside, nitrous oxide, food additives); nitrobenzene (used in lubricating oils, dyes), and many others [1,2].

We present a case of methemoglobinemia following topical lidocaine and benzocaine spray for transesophageal echocardiography.

## Case presentation

A 73-year-old African American male presented to the hospital for lower back pain and symptoms of urinary tract infection and a chronic great toe wound with discharge. His history was significant for type 2 diabetes mellitus, hypertension, and coronary artery disease, post-stenting five years ago.

His blood culture collected at the time of hospital admission was positive for *Streptococcus dysgalactiae*. Other significant laboratory findings at the time of admission were a complete blood count of 17000/mm^_3_^,_ _hemoglobin of 13 gm/dL, the random blood sugar of 212 mg/dL, blood urea/creatinine of 13/1.37 mg/dL, and raised inflammatory markers (erythrocyte sedimentation rate [ESR]: 140 mm/hr, c-reactive protein [CRP]: 299 mg/L). The patient was started on vancomycin and cefepime based on his blood culture report. Right hallux wound incision and drainage had *Streptococcus dysgalactiae*, *Staphylococcus capre*, and methicillin-resistant *Staphylococcus aureus*, then MRI confirmed right hallux osteomyelitis. He underwent right hallux amputation. Further workup showed that patient had a spinal abscess, which developed due to the seeding of bacteria from his toe infection. He underwent bilateral laminectomy and decompression with drainage of the abscess.

A transthoracic echocardiogram done to rule out infective endocarditis showed no vegetations. Due to high suspicion of infective endocarditis, transesophageal echocardiography was also performed using benzocaine 20% spray to the back of the throat before procedure initiation. In addition, the patient was given 1 mg of midazolam and 25 mcg of fentanyl for pre-procedure sedation. Within minutes after the procedure, the patient was cyanotic, diaphoretic, confused, and developed a facial droop on the operating table. Code Stroke was called. Vitals were blood pressure 99/56, heart rate 106, respiratory rate 30, saturating 76% on room air. He was started on supplemental O2 with a non-rebreather mask. He was intermittently bagged with saturation improving to 85% on 100% O2 with on and off improvement in mental status and facial droop resolved. The decision was made not to intubate the patient, and an arterial blood gas analysis (ABG) was done, which showed chocolate-brown colored blood and a partial pressure of oxygen (PaO2) of 185 mmHg with 70% methemoglobin (ABG-pH/PaO2/PaCO2/HCO3: 7.48/185/37/27.6). The patient was immediately given 1 mg/kg of methylene blue and transferred to the intensive care unit (ICU). Within 15 minutes, the patient was improving, with improved cyanosis, mental status, and improving diaphoresis. One hour repeat ABG showed methemoglobin levels of 31. Poison control was called, and they recommended methemoglobin levels less than 30, with a goal of less than 20, and resolution of symptoms. In the following hour, the attending toxicologist recommended re-dosing with another 1 mg/kg for a total of 2 mg/kg (100 mg) methylene blue for persistently elevated methemoglobin levels greater than the goal level. The repeat ABG after the second dosing of methylene blue showed methemoglobin levels less than 10. The toxicologist recommended following the methemoglobin levels on Arco oximetry ABG. His final ABG showed a methemoglobin level of 2.6 and saturation maintained with oxygen supplementation via nasal cannula. The patient was discharged the next day to a rehabilitation center with antibiotics.

We report this case to draw attention to rare circumstances of methemoglobinemia following the use of local anesthetics. This may not be considered and missed during code scenarios due to uncommon encounters, a general lack of awareness, and limited availability of standard co-oximetry for an accurate diagnosis.

## Discussion

A transesophageal echocardiogram (TEE) is used for better visualization of posterior cardiac structures not clearly visualized by transthoracic echocardiography. It is a safe procedure, and only a few significant complications have been reported. The major complications include esophageal perforation, gastrointestinal bleeding, pharyngeal hematoma, and methemoglobinemia [3-5].

Methemoglobinemia following TEE is due to local anesthetic spray use. In a retrospective study for all adverse events following benzocaine use, 132 of 198 cases (66.7%) were related to methemoglobinemia, which involved 107 serious events (81.1%) and even two deaths (1.5%). More than 90% of these cases were related to benzocaine spray use. Most of these cases of benzocaine-related methemoglobinemia happened in the inpatient setting following procedures needing endoscopy of any kind [6]. The incidence of methemoglobinemia was 0.067% in a series of over 28,000 TEEs performed in a center [7].

Risk factors for developing methemoglobinemia following medication use depends on several factors which affect the drug concentration (both levels and time taken to clear) in the blood: elderly, glucose 6-phosphate dehydrogenase (G6PD) deficiency, high doses, prolonged use, concomitant use of multiple oxidant drugs (lidocaine + benzocaine), route of administration, concomitant problems (heart, lung, liver, kidney), sepsis, and shock. Methemoglobinemia can present early or might take time to manifest after drug exposure (ranging from one to 10 hours). This is because some of the above medications need to transform into toxic metabolites before symptoms manifest [1].

Clinical presentation depends on the severity of methemoglobinemia. According to the increasing level of methemoglobin in the blood, the symptoms are cyanosis, chocolate-colored blood, headache, anxiety, tachycardia, dizziness, changes in mental status, tachypnea, and syncope arrhythmias, seizures, coma, and death [2]. Our patient presented with altered mental status, cyanosis, and hypoxia following TEE. Refractory hypoxemia, cyanosis, and chocolate-colored blood following any procedure or after the use of topical anesthetics are highly suggestive of methemoglobinemia.

The gold standard for diagnosis remains co-oximetry, but some blood gas analyzers give methemoglobin levels as well. Traditional pulse oximetry is unreliable in diagnosing methemoglobinemia. It cannot distinguish oxyhemoglobin from abnormal hemoglobin like methemoglobin, sulfhemoglobin, and carboxyhemoglobin which bind and carry oxygen to varying extents but do not release it to the tissues [2,8]. Oxygen saturation gap (difference between oxygen saturation measured by pulse oximeter and saturations by blood gas analysis) >5% indicates the presence of abnormal hemoglobin [9].

Treatment of severe methemoglobinemia with methylene blue is done only if the patient is symptomatic or methemoglobin >30% [6]. Care is needed with methylene blue as it is known to cause hemolysis in toxic doses or G6PD-deficient individuals [10-12]. However, most patients respond rapidly to the treatment, as seen in our patient.

## Conclusions

Topical anesthetic use is associated with methemoglobinemia. In addition, episodes of methemoglobinemia are reported after TEE. Therefore, it is crucial to keep methemoglobinemia very high in differential in cases of cyanosis, altered mental status, and hypoxia after application of local anesthetics. In addition, it is imperative to differentiate methemoglobinemia from other complications of TEE in such patients.
